# The Progressive Colonization of the Invasive Species *Amphistegina lobifera* on Pantelleria Island (Central Mediterranean, Sicily Channel)

**DOI:** 10.3390/biology14091282

**Published:** 2025-09-17

**Authors:** Claudia Cosentino, Antonio Caruso

**Affiliations:** Dipartimento di Scienze e Tecnologie Biologiche Chimiche e Farmaceutiche, Università degli Studi di Palermo, via Archirafi 18, 90123 Palermo, Italy; antonio.caruso@unipa.it

**Keywords:** benthic foraminifera, non-indigenous species, *Amphistegina lobifera*, marine biodiversity, Mediterranean Sea

## Abstract

*Amphistegina lobifera*, a symbiont-bearing benthic foraminifera native to the Indo-Pacific, is expanding its distribution range in the Mediterranean Sea due to *ysian* migration through the Suez Canal. This study investigates the colonization dynamics and ecological impact of *A. lobifera* on Pantelleria Island, which is located in the Central Mediterranean. Nine samples were collected from various substrates at different depths (−4 to −20 m) and analysed for composition, abundance, and ecological diversity. Results reveal a significant dominance of *A. lobifera*, especially in shallow unvegetated substrates, with relative abundances exceeding 50%. Conversely, more vegetated substrates host more diverse and balanced assemblages. Diversity indices are consistently lower where *A. lobifera* is dominant, indicating reduced biodiversity. Comparison with similar studies in Malta, Linosa, and Corfu confirms a 20% relative abundance threshold for *A. lobifera* as indicative of potential ecological imbalance. The results suggest that it represents a growing threat to native microbenthic biodiversity and reinforce its usefulness as a bioindicator species. Pantelleria represents a key transition zone for monitoring the dynamics of invasive species in the Mediterranean, and this study highlights the urgent need for long-term surveillance of benthic biodiversity.

## Simple Summary

In recent years, many marine species native to Indo-Pacific tropical areas have colonized the Mediterranean Sea, among them the benthic foraminifera *Amphistegina lobifera,* which lives on the seafloor and constructs a thick carbonate shell. This species arrived via the Suez Canal and has spread widely, partly due to the increase in sea surface temperatures recorded in recent years. In this study, we investigated how *A. lobifera* is spreading around the coasts of Pantelleria, a volcanic island located in the Sicily Channel. We collected seafloor samples at various depths and studied the microorganisms living both in sediments, algae, and on *Posidonia oceanica* meadows. We found that it is already highly present at most sampling sites, especially where marine vegetation is sparse or damaged. In places where *P. oceanica* meadows are well preserved, the number of native species is higher, and the presence of this invasive species is lower. The results suggest that *A. lobifera* is altering local biodiversity and the balance of the marine ecosystem.

## 1. Introduction

In the Mediterranean Sea, the phenomenon of *Lessepsian* migration, through the Suez Canal, is well documented in many organisms, such as fish, jellyfish, crustaceans, molluscs, soft corals, and algae [1,2,3,4,5,6,7]. In recent years, this migration has also been favoured by the progressive warming of the sea surface waters in the Eastern Mediterranean (Levantine Basin), which has made the environment more favourable for subtropical–tropical organisms from the Indo-Pacific [8,9]. In this context, numerous species of Indo-Pacific origin have successfully established in the Eastern Mediterranean and are gradually expanding towards the Central Mediterranean, but they are poorly represented in the Western Mediterranean. The term non-indigenous species (NIS) has been introduced to indicate organisms native to a specific geographic area that, through accidental or intentional causes, are introduced into ecosystems outside of their natural range. According to the definition provided by the International Union for Conservation of Nature 2000 [10], these are species that successfully establish in natural or semi-natural habitats, acting as agents of change and posing a threat to endemic biodiversity. Such species can compete with native species, becoming invasive and causing serious ecological imbalances, and are referred to as Invasive Alien Species (IAS).

Although the presence of NIS in the Mediterranean Sea is well documented, with over 1000 species [5], only in recent years has there been growing interest from the EU and local governments, with laws and implementing measures to regulate the various aspects related to their presence and to assess any potential damage to the marine environment. At the European level, the European Union’s Regulation on IAS No. 1143/2014 [11] represents the main regulatory framework [12]. This regulation establishes rules for the prevention and management of the introduction and spread of IAS, including marine ones. Among the NIS, some exotic species of benthic foraminifera have also been described [13,14,15,16]. Recently, Stulpinaite et al. [17] published an updated list consisting of 44 species of alien benthic foraminifera introduced via the Suez Canal pathway.

Foraminifera are unicellular marine microorganisms (planktonic or benthic) that may have a carbonate or arenaceous shell. Benthic species can be infaunal or epifaunal, the latter also including epiphytic forms. Foraminifera are essential organisms for the marine ecosystem as they constitute a fundamental link in the food chain and are widely studied as climatic, ecological, and environmental bioindicators [18,19,20]. The abundance of different species is closely linked to various environmental factors, better known as limiting factors. Among these, the most important are temperature, dissolved oxygen, pH, nutrients, turbidity, bathymetry, the presence of bottom vegetation, water transparency, etc. Finally, some polluting factors can influence assemblages by modifying specific diversity and association types [21,22,23,24,25,26].

Among benthic foraminifera, *Amphistegina lobifera* [27] is the most important exotic species considered as IAS. In particular, *A. lobifera* is a symbiont-bearing species, with a thick and robust carbonatic shell with dimensions up to 1–2 mm. Zmiri et al. [28] demonstrated in laboratory experiments that *A. lobifera* cease all movements when exposed to temperatures below 12 °C. Larsen, 1976 [27], reported that the occurrence of amphisteginids in modern oceans is delimited by the 14 °C winter isotherm [29,30]. Today, it is widely present in tropical and subtropical areas of the Indo-Pacific and the Red Sea [30,31]. It lives both as an epiphytic and epifaunal form and consumes an increasing concentration of oxygen as the temperature increases from 16 to 32 °C [32]. Furthermore, in tropical areas, it is well known for its ability to significantly contribute to the formation of biogenic carbonatic sands, which, from a sedimentological point of view, together with building corals, contribute to the formation of sands in the atolls [33,34]. Recently, Dämmer et al. [35] affirmed that *A. lessonii*, due to its high reproductive capacity for carbonate production, shows an optimum at slightly elevated pCO_2_ levels (700 ppm).

*Amphistegina lobifera* was first recognized in the Eastern Mediterranean in the early 2000s off the coasts of Israel, Malta, Turkey, Greece, Corfù, and Cyprus [13,36,37,38,39,40,41,42], in the islands of the Sicily Channel [14]. In recent years, it has reached increasingly higher percentages along the Mediterranean coasts, progressively migrating towards the west. Guastella et al. [15] described three stages of colonization for this species: (i) the early stage of colonization, with abundance values < 20%; (ii) the medium stage of colonization, with values between 20 and 50%; and (iii) the advanced stage of colonization, with values > 50%. A recent study [41] carried out by our team at Malta island, allowed us to predate the first appearance of *A. lobifera* during the Second World War, in 1943, with a strong increase starting from the 90s. *A. lobifera* is very abundant along the coasts of Greece [38,42], and it is spreading along the coasts of Albania [43], but to date, the low winter temperatures of the Adriatic Sea limit its spread towards the north. The progressive colonization of South-eastern Sicily has developed from an early to a medium stage of colonization, while it is well established with a medium-advanced stage of colonization in the islands located in the Sicily Channel, i.e., Pelagian and Pantelleria islands [14,15,16]. Instead, along the coasts of the Egadi Islands (the westernmost part of Sicily), it is still in the early phase of colonization, with percentages well below 5% [15]. In the Southern Tyrrhenian Sea and the Western Mediterranean, it has not yet been reported [14]. The aim of this work is to first verify the colonization process of *A. lobifera* on Pantelleria Island in comparison with the data reported by Guastella et al. [15], and second, to evaluate the impact on foraminiferal biodiversity. Pantelleria Island is located in the centre of the Sicily Channel, and its geographical position between the Western and Eastern Mediterranean makes it an ideal site for marine biology studies, as well as useful for monitoring the progressive colonization of organisms from the Indo-Pacific. The presence of different NIS reflects the environmental fluctuations to which the water masses of the Sicily Channel are subject and the interactions between species of Atlantic affinity from the Western Mediterranean and those of the Indo-Pacific.

## 2. Study Area

### 2.1. Geological and Environmental Setting

**Figure 1:**
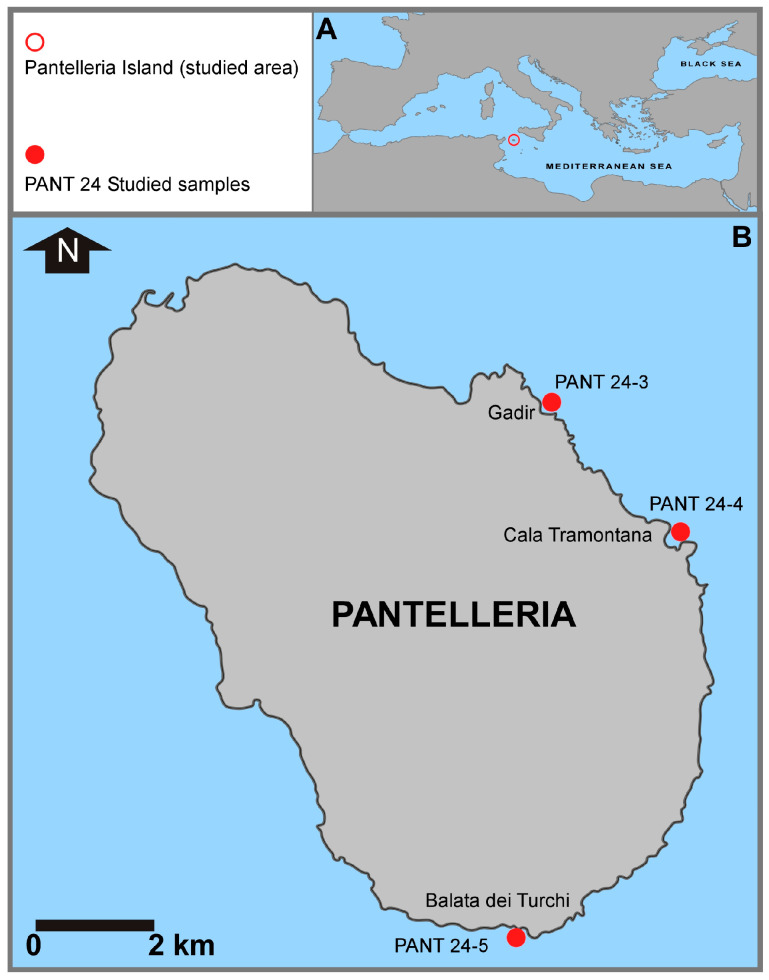
(**A**) Location map of the studied area in the Sicily Channel (Mediterranean Sea) and (**B**) location of the sampling stations around the island of Pantelleria.

The island of Pantelleria is located in the central sector of the Sicily Channel (Central Mediterranean, Figure 1), approximately 110 km southwest of the Sicilian coast (Cape Granitola) and 70 km northeast of the coast of north Africa (Cape Kelibia, Tunisia). Pantelleria is elliptical in shape, extending in a NW-SE direction, and is the largest of the islands in the Sicily Channel, approximately 80 km^2^ and 51 km of coastline.

From a geographical and geological point of view, it represents a unique environment characterized by a complex geology, with predominantly volcanic rocky coasts and a narrow, jagged continental shelf. This island has been called the “Black Pearl” of the Mediterranean because it is entirely of volcanic origin, and therefore, its landscape is typically dark in colour. Pantelleria represents the emerging tip of a complex volcanic edifice, mostly submerged, which reaches a depth of approximately 1200 m and is located in the axial part of the Sicily Channel Rift. The first submarine volcanic activity that gave rise to the island dates back to approximately 1 Ma. The island emerged stably around 300 ka with the onset of the first explosive subaerial volcanic activity. The oldest volcanic rocks of Pantelleria have an age of approximately 324 ka [44] and vary in composition from weakly alkaline basalts to trachytes and rhyolites with peralkaline affinity [45]. Pantelleria is dominated by Montagna Grande, a relief 836 m above sea level, which is a volcanic caldera formed approximately 45 ka [44,46]. From a lithological point of view, the most common rocks are pantellerites, basalts, green tuff, and pyroclastic deposits. One of the most recent eruptions occurred in 1831 in an underwater environment, north-west of the island, and created a small island known as Isola Ferdinandea [47]. This remained above sea level for only eight months and then disappeared completely in 1832, forming a vast rocky platform (Graham Bank).

Today, it lies eight meters below sea level and is a rich fishing area, recognized as a biodiversity hotspot thanks to the presence of many protected pelagic and benthic marine species. Pantelleria Island is protected as “Pantelleria Island National Park,” established in 2016 and covering 80% of its territory. The park covers only the land area, not the marine area. It constitutes a complex ecological system characterized by significant biodiversity, both terrestrial and marine. The marine–coastal environment, characterized by volcanic rocky substrates, *Posidonia oceanica* meadows, marine algae, and, more rarely, small sandy areas, provides an ideal habitat for a wide variety of marine organisms. Here, the marine environment is characterized by an excellent degree of transparency of the waters, as reported at Linosa Island by Cosentino et al. [16]. Transparency permits light to reach deeper depths, allowing symbiont-bearing organisms to extend their habitat.

Furthermore, the waters surrounding the island are influenced by the interaction between Atlantic and Levantine waters, giving the area particularly dynamic oceanographic characteristics. The local marine climate is typically warm–temperate, but in recent decades, it has shown signs of tropicalization, consistent with trends observed in other areas of the Southern Mediterranean. Generally, in recent years, the sea surface temperature (SST) of the Central Mediterranean, between Sicily and North Africa, fluctuates between 15 °C and 29 °C during the year [48,49].

In particular, South-western Sicily SSTs are characterized by more or less stable low temperatures during the winter (15–16 °C), while they are characterized by wide oscillations during the summer (17–26 °C). However, in the South-eastern part of Sicily and in the Sicily Channel (around Pantelleria, the Pelagian Islands, and Malta), average summer temperatures are higher and more stable with smaller oscillations. The marine algal flora shows strong North African affinity [50,51,52,53,54].

### 2.2. Exotic Species at Pantelleria

Terrestrial biodiversity has been the object of numerous studies describing the presence of exotic species [55,56,57,58,59,60,61]. Regarding exotic marine organisms, several species have been described, such as *Rhopilema nomadica* [62], *Portunus segnis* [63], the algae *Caulerpa cylindracea* and *Caulerpa taxifolia* [64], and *Parupeneus forsskali* [65]. On the contrary, regarding marine microorganisms (i.e., benthic foraminifera), the work of Guastella et al. [15] is the only study focused on benthic foraminiferal assemblages in this island. In particular, these authors reported the presence of NIS and several cryptogenic species. They recorded *A. lobifera* at all four study sites (sampled in 2017) with abundances ranging from 2% to 82%, even if, in that study, the authors reported only the total assemblages, not discriminating between dead and living specimens. Thus, the percentages were probably overestimated, hypothesizing a stage of colonization from early to advanced. In this study, we provide a better evaluation.

## 3. Materials and Methods

**Figure 2:**
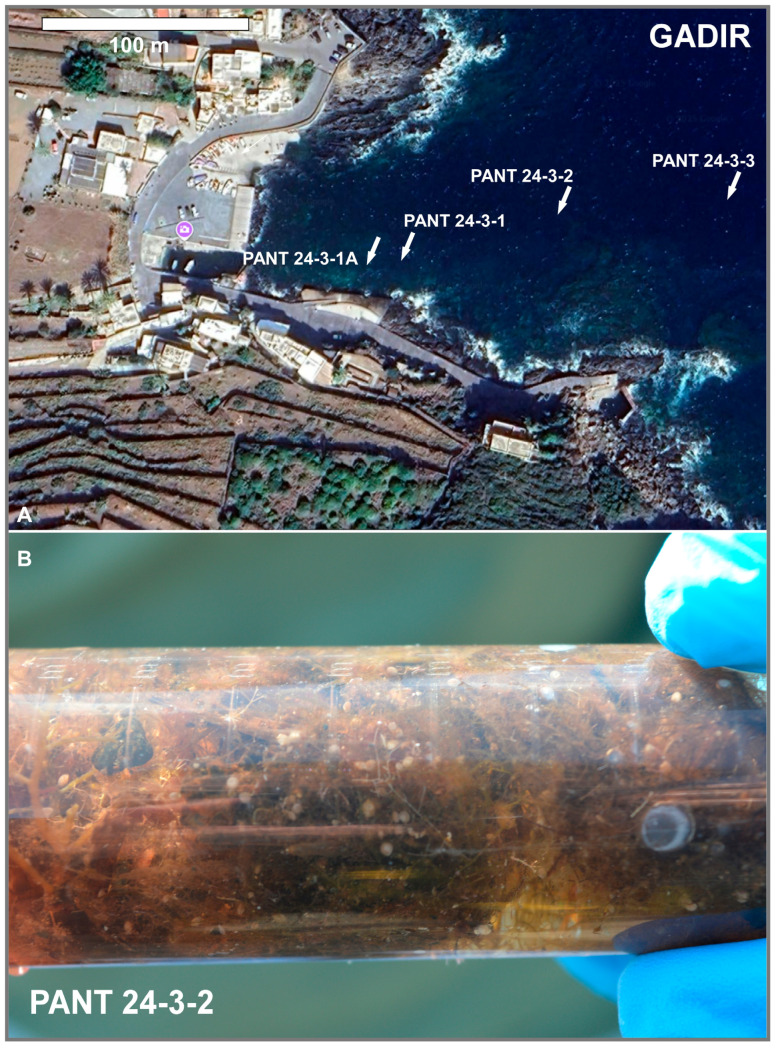
(**A**) Location of samples collected at the Gadir site (image from Google Earth); (**B**) detail of the PANT 24-3-2 sample stored in a cylindrical polyethilene container (Falcon). All the white dots visible to the naked eye are shells of living *Amphistegina* attached as epiphytes to algae.

**Figure 3:**
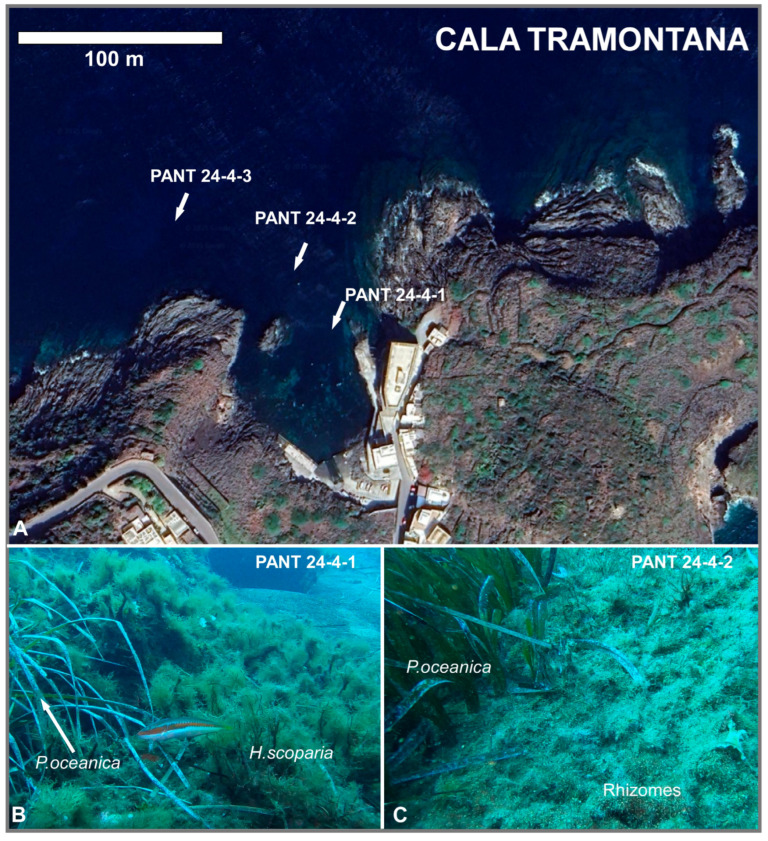
(**A**) Location of samples collected at Cala Tramontana (image from Google Earth). (**B**) PANT 24-4-1 (−7 m); *Posidonia oceanica* and the brown algae *Halopteris scoparia* are clearly visible on the rocky substrate. (**C**) PANT 24-4-2 (−9 m); particularly for *P. oceanica*, on the right, only rhizomes were found, since the meadow was destroyed by touristic boat anchoring in summer. The rest of rhizomes are rich in *Amphistegina* shells (white dots).

**Figure 4:**
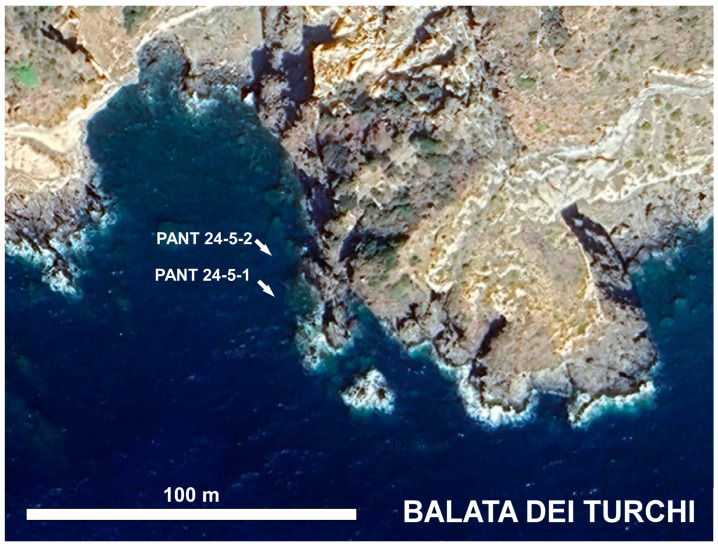
Location of samples collected at Balata dei Turchi (image from Google Earth).

In October 2024, scuba diving expedition was conducted at Pantelleria (Italy) to collect samples from three sites (Gadir, Cala Tramontana, and Balata dei Turchi, respectively; Figure 1, Figure 2, Figure 3 and Figure 4).

Algal and/or sediment samples were collected at each site at different depths. A total of nine samples were collected. Table 1 reports the geographic coordinates of the sampling stations, depths, and the type of sample collected. SSTs and salinity were also measured (Table 1). Underwater photographs were taken with a GOPRO 8 camera (GoPro Inc., San Mateo, CA, USA).

The samples were collected at depths between 4 and 20 m, divided into four aliquots, and stored in cylindrical polyethilene containers. One aliquot was preserved for possible sedimentological and/or geochemical analyses. Another aliquot was treated with a buffered Bengal Rose solution (2 g of Bengal Rose in one litre of ethanol) to differentiate living (coloured) benthic foraminifera from those already dead (uncoloured) at the time of sampling [66]. Two aliquots of each sample were also treated with two different chemical products, PowerProtect DNA/RNA reagent QIAGEN (Hilden, Germany) and RNAlaterTM Solution Invitrogen by Thermo Fischer Scientific (Vilnius, Lithuania) to preserve both DNA and RNA and subsequently carry out genetic studies on living foraminifera.

The samples were stored in a cool bag, then transported to the laboratory and refrigerated until ready for analysis.

The algal species were identified in situ and subsequently verified at the laboratory for taxonomic identification.

Following the FOBIMO protocol [67], after 14 days, each Bengal Rose-treated sample was removed from the polyethilene container, gently washed on a 63 µm mesh sieve, and placed in an oven to dry for 24 h at 40 °C. The algal samples were prepared by carefully removing any benthic foraminifera attached to the algae or *P. oceanica* rhizomes. The samples were then placed in small plastic containers, labelled, and stored for further microscopic analysis.

In the first phase, a qualitative analysis was performed on the samples using a WILD HEERBRUGS binocular microscope, and the benthic foraminiferal species present were recognized and classified following [31,68,69,70]. Regarding the genus *Amphistegina*, the presence of a particular morphotype was observed, similar to *A. lessonii* but characterized by a very small size, a very flattened spiral side, and a pronounced carina, which was identified, according to Cosentino et al. [16], as *Amphistegina* morphotype alfa. This morphotype also corresponds to *Amphistegina* cf. *lessonii*, first reported in Caruso and Cosentino [14]. The genus *Amphistegina* was photographed both by using a Leica optical system with DFC420 camera (GoPro Inc., San Mateo, CA, USA) and an SEM PHENOM PROX (Thermo Fisher Scientific, Segrate, Italy).

Subsequently, quantitative analyses were performed on benthic foraminifera. Each sample was split into smaller aliquots using an Otto microsplitter produced by Green Geological (Whittier, CA, USA). The split fraction was weighed, and all the individuals present were counted, distinguishing those alive at the time of sampling (coloured) from those dead (uncoloured).

To evaluate the benthic foraminiferal community, four diversity indices were calculated using Paleontological Statistics Data Analysis (PAST) software v4.03 [71]: (1) species richness (S), i.e., the number of species in each sample; (2) dominance (D), an index ranging from 0 to 1 that indicates how single species are distributed within the assemblages; (3) the Shannon index (H), which measures both the richness (number of species) and the evenness (distribution of abundances) of a biological community; and (4) the Fisher-α index, i.e., the relationship between the number of species and the number of individuals in an assemblage [72,73].

## 4. Results

In the studied samples, the assemblages are dominated by living foraminifera, and the dead specimens constitute only a small percentage of the total assemblage (Table 2). In some cases, the assemblages were only characterized by living specimens (PANT 24-3-1A, PANT 24-3-1 and PANT 24-3-2). Moreover, in the other samples, dead specimens constitute less than 6% of the total assemblage, except for the samples PANT 24-3-3 and PANT 24-5-2, 20.81% and 27.93%, respectively. For this reason, we decided to focus our attention on the living assemblages.

A total of 30 species belonging to 21 genera were recognized. In Table 3, we report only the living benthic foraminiferal species (29), not including *Elphidium* sp., as only one dead individual was found in the studied samples.

**Figure 5:**
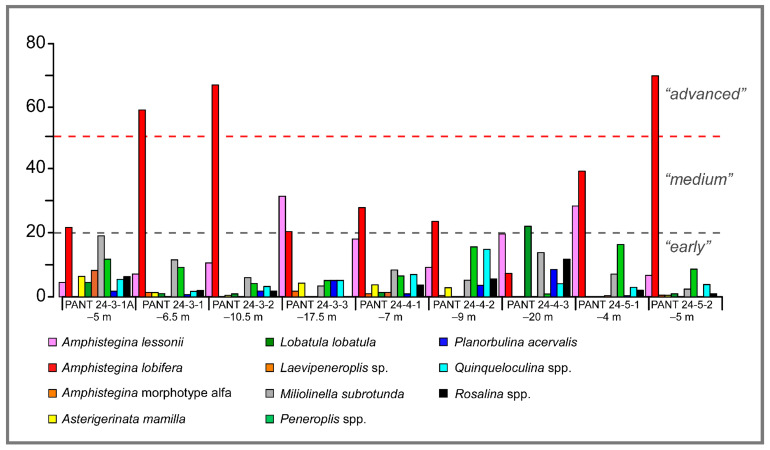
Relative abundances of living benthic foraminifera in samples from Pantelleria Island. *A. lobifera* reaches percentages >20% except in the sample at −20 m depth. In the deeper samples, *A. lessonii* is more abundant with respect to *A. lobifera*.

Relative percentage abundances of all the living species recorded are reported in Table 3 as numerical values, while the most relevant species are reported as histograms in Figure 5. The analysis of the association highlights an overall community dominated by a small number of species, with strong variability between samples in relation to depth and type of substrate. Among the most abundant species, *A. lobifera* stands out, with percentages ranging between 7.3% (PANT 24-4-3, −20 m) and 69.9% (PANT 24-5-2, −5 m). Its dominance is particularly marked in samples with shallower bathymetries. *Amphistegina lessonii* is present in all samples, with percentages ranging from 4.5% to 31.4% (PANT 24-3-1A, −5 m), and is abundant at deeper depths. *Amphistegina* morphotype alfa is present in only five samples, with very low percentages, ranging from 0.4% (PANT 24-4-2, −9 m) to 1.69% (PANT 24-3-3, −17.5 m).

Among the native and common species in the Mediterranean Sea, miliolids are always present. Among these, *Miliolinella subrotunda* shows significant percentages in almost all samples (minimum value of 2.4% at PANT 24-5-2 and maximum value of 18.9% in PANT 24-4-3). *Peneroplis pertusus* is present in eight samples, with values ranging from 3.2% at PANT 24-3-2 to 16.2% in PANT 24-5-1. *Laevipeneroplis* sp. is present in only three samples, with the highest percentage at PANT 24-3-1A (8.1%). Individuals belonging to the genus *Quinqueloculina* are characterized by variable abundances that, however, never exceed 7.5%.

*Textularia pala*, a foraminifer with an agglutinant shell, is present in all samples, with percentages ranging from 1.4% at PANT 24-3-2 to 16.1% at PANT 24-3-3.

*Rosalina obtusa* and *Asterigerinata mamilla*, typical epiphytic species, are almost always present, but with percentages below 6.3% and 11.7%, respectively. Other species, such as *Adelosina* sp., *Lobatula lobatula*, *Planorbulina acervalis,* and *Vertebralina striata*, are present only at particular sites with highly variable abundances.

**Figure 6:**
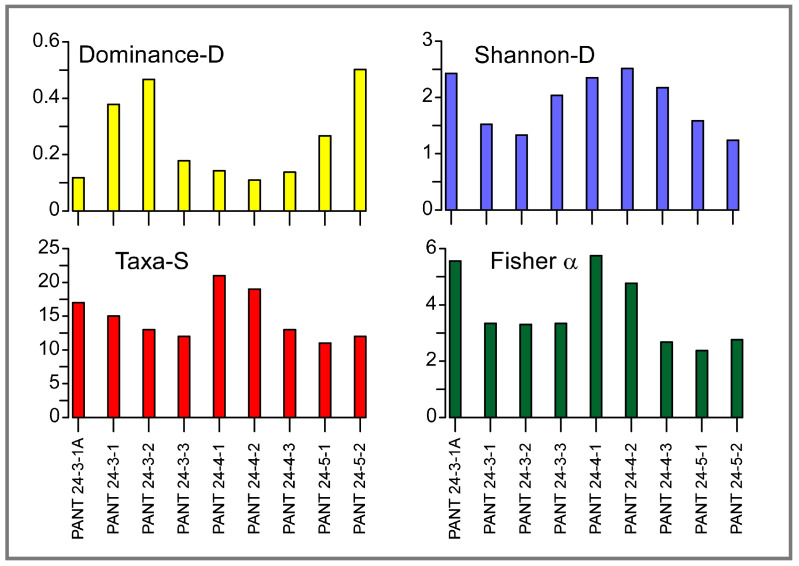
Diversity indices calculated for living benthic foraminiferal assemblages.

The samples were also analysed to assess the diversity and structure of the foraminiferal community. The results obtained using PAST software highlight some variability among samples in terms of species richness (S), dominance (D), Shannon index (H′), and Fisher’s α (Figure 6).

The number of taxa (species richness, S) ranges from 11 to 21 species, with the highest values found in samples PANT 24-4-1 (S = 21) and PANT 24-4-2 (S = 19), suggesting a more diverse community. In contrast, samples PANT 24-5-1 and PANT 24-5-2 show the lowest species richness (S = 11 and 12, respectively). The dominance index (D), which expresses the degree of dominance of a single species, ranges from 0.109 to 0.502. The lowest values (D ≈ 0.11–0.14) correspond to samples PANT 24-4-2, PANT 24-4-1, and PANT 24-3-1A and indicate more evenly distributed communities, while for the PANT 24-5-2 sample (D = 0.502), Shannon index (H′) values range between 1.237 and 2.512, with the most diverse samples always corresponding to those with low dominance (i.e., PANT 24-4-2, H′ = 2.512). Samples with H′ < 1.6 (e.g., PANT 24-5-2 and PANT 24-3-2) instead show low diversity, with an association dominated by a few taxa.

Finally, Fisher’s α index shows values between 2.38 and 5.75, confirming the greater specific heterogeneity of the PANT 24-4-1 (α = 5.75) and PANT 24-3-1A (α = 5.60) samples and a simpler structure in the samples characterized by high dominance.

Overall, the samples on algal substrates show greater diversity and a more even distribution, while the sediment sample PANT 24-5-2 is characterized by lower diversity and is dominated by a few high-dominance species (D = 0.502).

## 5. Discussions

### 5.1. Benthic Foraminiferal Assemblages and Diversity Indices

Benthic foraminiferal analysis highlights an assemblage dominated by a limited number of species, with a distribution strongly influenced by substrate, depth, and the presence of vegetation, dominated by the genus *Amphistegina*. In particular, *A. lobifera* is the dominant species, which is widely represented in almost shallow sites, with values exceeding 50%, essentially attached as an epiphyte to the *Posidonia oceanica* rhizomes and *Halopteris scoparia*, and in the sandy substrate as an epifaunal species. *A. lobifera* is associated with *A. lessonii* and *Amphistegina* morphotype alfa, albeit in lower percentages. The highest percentages of *A. lobifera* occur in samples with bathymetries less than 11 m and in substrates where *P. oceanica* is strongly degraded due to the high number of summer anchorages that damage the sites where there is a high summer tourist influx. This type of damage also facilitates predation by the fish species *Sarpa salpa* and the echinoderm *Paracentrotus lividus*, which feeds on *P. oceanica*. In these cases, the predominance of *A. lobifera* corresponds to a drastic reduction in species diversity, highlighted by ecological indices (H′ and Fisher’s α), suggesting a significant ecological impact on the local foraminiferal assemblage. Conversely, in samples with deeper bathymetries, the species’ abundance is lower, and the foraminiferal assemblage is more balanced, presumably also favoured by greater variability of plant and algal species. In these environments, *A. lobifera* coexists with other species without significantly altering the overall composition of the assemblage. *Amphistegina lessonii* is present in all samples with variable percentages, suggesting a possible competitive coexistence with both *A. lobifera* and the morphotype alfa.

**Figure 7:**
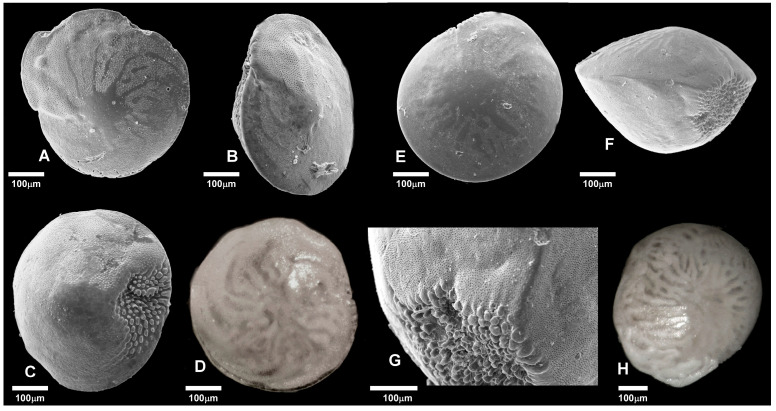
(**A**) *A. lessonii*, spiral side (PANT 24-3-3); (**B**) *A. lessonii*, lateral view; (**C**) *A. lessonii*, ventral side; (**D**) *A. lessonii*, spiral side; (**E**) *A. lobifera*, spiral side (PANT 24-3-2); (**F**) *A. lobifera*, lateral view; (**G**) *A. lobifera*, detail of the aperture; (**H**) *A. lobifera*, spiral side (PANT 24-3-3). (**A**–**C**,**E**–**G**): SEM photographs, (**D**,**H**): optical microscope photographs.

In particular, *A. lessonii* (Figure 7, photos A, B, C, D) is one of the most abundant species in the algal samples collected at deeper bathymetries (31.4% in PANT 24-3-3 and 19.6% in PANT 24 4-3, at −17.5 and −20 m depth, respectively; Figure 7, photo A).

This pattern suggests that *A. lessonii* tends to be dominant in similar but deeper habitats and tends to occupy complementary niches. The presence of *Amphistegina* morphotype alfa is marginal, with percentages ≤1.7%, indicating a presence with no significant impact on the structure of the assemblages. From a taxonomic point of view, this morphotype cannot be clearly classified, and therefore, it requires greater attention through genetic analysis. This will be further developed in the future, also thanks to the 18SrDNA analyses currently being conducted at our laboratories.

Diversity indices reveal patterns consistent with the literature regarding foraminiferal community. Diversity indices highlight considerable variability in foraminiferal community structure, likely reflecting the different environmental characteristics of the substrates. The living benthic foraminiferal assemblage shows high variability in terms of dominance and species richness, with a clear distinction between samples collected on vegetated substrates and those on sandy sediments.

At some sites, dominance highlighted the presence of a few species characterized by high abundance, indicating less structured communities and dominated by opportunistic taxa, including *A. lobifera.* Conversely, high values of the Shannon index and Fisher’s α in other samples reflect more diversified and uniformly distributed communities, suggesting more stable and/or less disturbed environments. Samples taken from vegetated substrates show high values of species richness, Shannon index, and Fisher’s α, which are associated with low dominance. This suggests that the presence of vegetation contributes to creating a more stable and complex microhabitat capable of supporting a more diverse and balanced community. Overall, samples collected from sites with abundant vegetation show a more heterogeneous benthic foraminiferal community, with abundances distributed among different species, while samples on sandy substrates without vegetation are characterized by a high dominance of *A. lobifera* (Figure 7, photos E, F, G, H), accompanied by an overall reduction in diversity indices. In contrast, the sediment sample PANT 24-5-2 (−5 m) is characterized by strong dominance (D > 0.5), low diversity (H′ < 1.3), lower species richness (S = 12), and reduced Fisher’s α values, indicating an ecologically simplified community. In particular, the abundant presence of *A. lobifera* in this latter sample (69.86%) is indicative of the competitive and colonizing capacity of this non-native species, whose expansion in the Eastern and Central Mediterranean Sea is now well documented [14,15,16,74].

This pattern confirms the structuring role of the substrate and the invasiveness of the *Lessepsian* species in the organization of the benthic foraminiferal communities. Less stable sedimentary conditions, devoid of vegetation, subject to wave energy, and highly transparent waters that favour the penetration of sunlight, facilitate the dominance of *A. lobifera.* As previously reported, it is an opportunistic symbiont-bearing species, highly adaptable, which tends to reduce community complexity and modify the microbenthic trophic chain. Conversely, vegetated substrates offer shade, stability, and greater roughness, elements that favour the coexistence of a large number of taxa and partially prevent the expansion of invasive taxa. In some sites, boat anchoring during the summer months causes a high level of deterioration of *P. oceanica* meadow, which triggers an increase in leaf predation, leaving the rhizomes exposed, which are promptly colonized by *Amphistegina.*

The abundance of *A. lobifera* in well-lit waters and poorly vegetated sand substrates is consistent with data described in the Pelagian and Malta Islands, where they dominate surface sediments, actively contributing to biogenic carbonate [14,15]. During scuba diving and sampling, we observed that *A. lobifera* is less abundant where the *P. oceanica* meadow is well developed without deterioration. In particular, in samples where there was good vegetation, higher species equity has been observed, with an enrichment in the abundance of *Miliolinella subrotunda*, *Peneroplis pertusus*, *Quinqueloculina* spp., and *Rosalina obtusa*. This distribution suggests that the presence of benthic macrophytes acts as a stabilizing factor capable of limiting the dominance of invasive species and promoting more diverse communities. Conversely, in unstable or more degraded environments, ecological competition is unbalanced in favour of *A. lobifera*, whose high abundance is also linked to a reduction in diversity indices and the alteration of the benthic foraminiferal assemblages [16,75]. Furthermore, it is important to note the presence of *Textularia pala* in all samples, a foraminifer characterized by an agglutinating shell. The genus Textularia is generally reported in the literature, in the Mediterranean Sea, as a typical form found in sandy and detrital bottoms in the infralittoral and circalittoral zones [76] but also as an epiphytic species *in P. oceanica* rhizomes [77]. In our samples, it is always present and abundant, probably due to the high energy of the marine environment, which suspends minerals and clasts derived from volcanic rock erosion, which are used to build the agglutinant test of Textularia.

The results indicate that algal vegetation acts as an ecological buffer, supporting more balanced benthic foraminiferal communities in the presence of *P. oceanica* meadows or macroalgae (PANT 24-3-3, PANT 24-4-3), while less structured substrates favour the dominance of *A. lobifera*. This is consistent with observations in other areas of the Mediterranean, where species prevalence above 20% is associated with a decline in the specific diversity of benthic foraminifera [16].

These findings confirm that benthic foraminiferal diversity can be used as a sensitive bioindicator to assess the ecological status of benthic seabeds and to monitor the impact of IAS in Mediterranean coastal environments, including islands like Pantelleria. In Pantelleria, *A. lobifera* percentages exceed 20% in eight of nine samples, a threshold considered in the literature to be a potential indicator of ecological imbalance in coastal environments [16,75].

This suggests that the island of Pantelleria, although characterized by a still complex and diverse benthic habitat, is already affected by active bioinvasion of *A. lobifera*.

### 5.2. Colonization of A. lobifera in the Sicily Channel

The expansion of *A. lobifera* in the Mediterranean is well-documented, with differential impacts on the structure of benthic communities depending on the relative presence of the species, the nature of the substrate, and bathymetry. The results obtained fit coherently with the colonization of *A. lobifera* in other sites of the Eastern Mediterranean Sea, a *Lessepsian* species now widely documented in shallow marine environments in the eastern and central sectors of the basin. The previous studies carried out in the Pelagian Islands and the eastern Aegean Sea have highlighted similar ecological patterns. In the Pelagian Islands, *A. lobifera* was reported by Cosentino et al. [16] as one of the most abundant species in sandy surface sediments, where it tends to replace the other species, particularly in sites with low vegetation coverage. Sediments studied around Linosa island show a marked sedimentary “switch,” from a dark volcanic matrix to almost entirely white biogenic sediments due to the accumulation of calcareous shells of *A. lobifera*. This process has led to the formation of environments similar to those observed in Indo-Pacific atolls, where this species is known for its ability to produce large-scale carbonate sediments. This phenomenon has also been observed at Pantelleria, although this island does not favour the formation of sand beaches along the coastline due to the particular geological conformation, where biogenic sand accumulations can occur a few meters below the sea level. In fact, in areas where the *P. oceanica* meadow is severely degraded, significant accumulations of *Amphistegina* shells are forming above the rhizomes, creating biogenic sand. In the Aegean Sea, several authors [38,75] described that the massive presence of *A. lobifera* is strongly correlated with sandy substrates and well-lit waters, with critical dominance values observed starting from thresholds >20%. In particular, Weinmann et al. [75] proposed an ecological threshold of 20% in samples off of Corfù (Greece), that is, the relative abundance beyond which a statistically significant effect on the diversity and balance of benthic foraminiferal communities is observed. In detail, this study demonstrated that *A. lobifera* values above 20% are associated with a significant reduction in the Shannon index (H′), an increase in the dominance index (Berger–Parker), and a partial reduction in Fisher’s α and species richness, although less marked.

The data obtained at Pantelleria are fully consistent with these observations. In eight of the nine analysed samples, *A. lobifera* exceeds the threshold of 20%, with a peak of 69.86% in sample PANT 24-5-2 (sedimentary substrate). In these samples, H′ values were lower than 1.6, consistent with strong taxonomic dominance, dominance (D) values up to 0.50, and low Fisher’s α, confirming the pressure exerted by the species, especially in the absence of vegetated substrates. In samples with a presence lower than 20% (i.e., PANT 24-4-3, 7.3%), diversity indices were more balanced, and the benthic community maintained a higher level of diversity, confirming the important role played by algal and vegetated substrates.

This convergence between the data from Corfù and Pantelleria supports the hypothesis that the 20% threshold value represents a reliable indicator of potential ecological imbalance linked to the invasiveness of *A. lobifera* and suggests the usefulness of this parameter as an early warning criterion in benthic biomonitoring programs in Mediterranean areas subject to colonization by *Lessepsian* species.

By comparing data from Pantelleria, the Pelagian Islands, and the Aegean Sea, it is clear that *A. lobifera* responds consistently to the same favourable environmental conditions, displaying synergistic invasive behaviour throughout the Central–Eastern Mediterranean basin. Its progressive expansion along the south-western coasts of Sicily and in the Sicily Channel clearly demonstrates the process of “tropicalization” of the Mediterranean, triggered by the opening and widening of the Suez Canal, as well as by the increase in Mediterranean SSTs over the past 30 years, especially in the Eastern Mediterranean.

Since 1993, ocean SSTs have increased globally by an average of more than 0.4 (±0.02) °C, while in the Mediterranean Sea, they have increased by about 1.5 °C (±0.02) [49]. In this context, Pantelleria represents an ecological transition area of particular interest, where the coexistence of indigenous and *Lessepsian* species reflects ongoing dynamics of competition and adaptation. Long-term monitoring of benthic foraminiferal communities and assessment of the impact of the *A. lobifera* invasion are therefore essential to understanding the evolutionary trajectory of these ecosystems. The comparison between Pantelleria and Mediterranean sites highlights how, despite the island’s geological and environmental peculiarities (volcanic substrates, variable depths, and the presence of photophilous macroalgae), the patterns of microhabitat dominance and preference of *A. lobifera* are replicable and comparable with other areas of the Central and Eastern Mediterranean. Favourable habitats characterized by shallow depth, incoherent sediments, and water transparency make the area vulnerable to the persistence and potential expansion of this species. At the same time, the presence of benthic macrophytes (i.e., *P. oceanica*) and the structural complexity of some habitats appear to exert a stabilizing effect, favouring more heterogeneous communities and reducing the absolute dominance of *A. lobifera*, as also observed in Linosa and Corfù islands.

**Figure 8:**
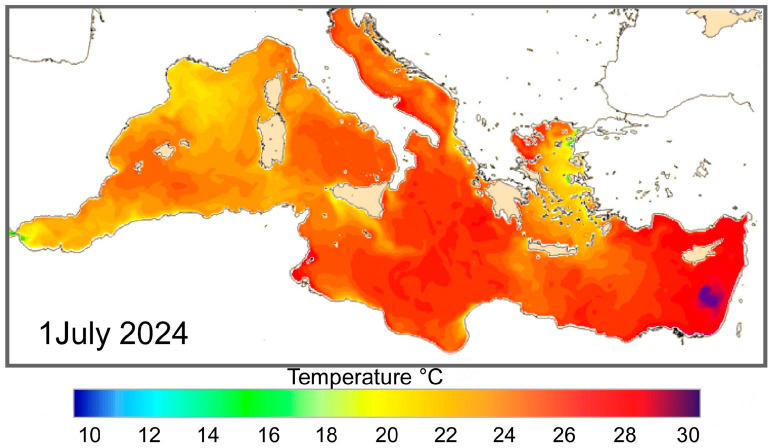
An example of the sea surface temperatures (in Celsius degrees) in the Mediterranean Sea recorded on 1 July 2024 (data from https://data.marine.copernicus.eu/, accessed on 28 July 2025). Off the coast of Southern Sicily, the temperatures are lower with respect to Sicily Channel.

**Figure 9:**
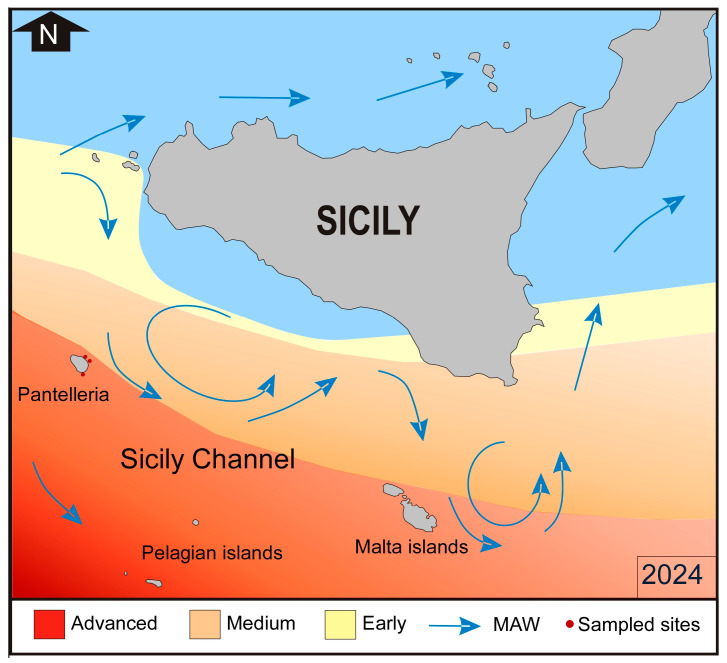
Schematic roadmap of the surface water circulation in the Sicily Channel (blue arrows) modified from [15]. The different colours indicate the stage of colonization of *A. lobifera*, from early (yellow) to advanced (red). The degree of colonization of *Amphistegina* is controlled by SST as reported in Figure 8.

The areal distribution and abundance of *Amphistegina* closely match the surface temperatures recorded on the Copernicus website [48]. The Atlantic waters feed the Mediterranean Sea, and during the winter, the lowest temperatures range between 15 and 16 °C, whilst reaching the highest temperatures (~23 °C) during the summer [49]. SSTs increase eastwards, especially during the summer, whilst in the Sicily Channel, they remain more or less constant during the winter [49,78]. The areas around Southern Sicily are characterized by strong summer temperature variations linked to current circulation and atmospheric conditions. Mistral storms cause upwelling of cooler water, likely limit the distribution of *Amphistegina* during the summer, and, to date, prevent their spread and colonization in the Tyrrhenian area. Although they can tolerate temperatures around 14–15 °C, they are unable to proliferate at lower temperatures. Throughout the year, temperatures around Pantelleria Island range from 15 °C to 28–29 °C, with higher and more stable average values during the summer compared to the coasts of Southern Sicily. The higher temperatures (Figure 8) favour their proliferation, which reaches an advanced stage of colonization (Figure 9).

Biological invasions of IAS are currently one of the main drivers of habitat degradation and a major cause of biodiversity loss in both marine and terrestrial ecosystems. In particular, in the Mediterranean Sea, the spread of invasive species of *Lessepsian* origin poses a particularly serious environmental threat to small islands that are highly vulnerable to biodiversity loss. For this reason, several European directives have been enacted to address this issue. The Marine Strategy Framework Directive (MSFD) 2008/56/EC [79] considers the presence of non-native species as one of the 11 descriptors of environmental quality (Descriptor 2: “Introduced non-native species”). The Directive requires Member States to ensure that “the introduction of non-native species is minimized and that these species do not cause negative impacts on marine ecosystems.” Furthermore, Regulation (EU) No. 1143/2014 [11] states that “After the introduction of an invasive alien species, early detection and rapid eradication measures are crucial to prevent their establishment and spread”. In Italy, the implementation of these regulations has led to the development of monitoring plans made by the Regional Agency for Environmental Protection (ARPA), as well as the Higher Institute for Environmental Protection and Research (ISPRA) and the collection of data through networks such as the International Commission for Scientific Exploration of the Mediterranean (CIESM) or the European Alien Species Information Network (EASIN).

In addition, to complement traditional monitoring efforts, citizen science is increasingly being adopted. In this context, the use of Local Ecological Knowledge (LEK) has proven effective for tracking non-native species [80].

The main problem is that many species are also commercially exploited, and it is difficult to initiate an eradication process, while on the other hand, it is even more difficult, if not impossible, to eradicate microorganisms such as foraminifera, i.e., the genus *Amphistegina*. Their spread, coupled with rising temperatures, may pose a serious threat to the future of typical Mediterranean species in the coming decades. On the other hand, the increase in abundance of *Amphistegina* in the Mediterranean could favour the production of carbonate shells in sea surface waters, counteracting the acidification processes due to the increase in CO_2_ in the atmosphere. Thus, we are witnessing a true biological revolution, with ecosystems undergoing the fastest changes after the last glacial maximum.

## 6. Conclusions

This study confirms the progressive colonization of *Amphistegina lobifera* around the coasts of Pantelleria Island (Sicily Channel), providing new data on its environmental preferences and its impact on benthic foraminiferal communities. Living benthic foraminiferal assemblages highlighted the increasing impact of *A. lobifera*, whose ecological success appears to be favoured by local environmental conditions, such as, for instance, the degradation of *P. oceanica* meadows, incoherent sediments, well-lit waters, and substrates with little vegetation. The anticorrelate behaviour between high abundances of *A. lobifera* and low biodiversity indices (Shannon index, Fisher’s α, and species richness) highlights its potential role as a factor in ecological imbalance in microbenthic communities. In particular, sedimentary substrates devoid of vegetation are more vulnerable to invasion, while habitats with algae and *Posidonia oceanica*, where the associations exhibit greater equality and a significant representation of native species, appear to mitigate the dominance of this invasive species, promoting more diverse and stable associations. The presence of *A. lobifera* in all the study sites, with abundances exceeding the ecological threshold of 20%, indicates that at Pantelleria is no longer in the initial phase of colonization, but rather in an intermediate or advanced stage. Comparison with other Mediterranean contexts (i.e., the Pelagian Islands and the Aegean Sea) confirms that the dynamics observed here are part of a broader process of tropicalization of the Mediterranean Sea, triggered by the opening of the artificial Suez Canal and favoured by maritime traffic and the progressive warming of Eastern Mediterranean surface waters that facilitated the migration of Indo-Pacific organisms.

In this context, *A. lobifera* is confirmed as an effective bioindicator of ongoing changes, both from an ecological and sedimentological point of view, as it causes significant changes in both local endemic biodiversity and coastal sedimentation.

Thus, the island of Pantelleria represents a biogeographical frontier area of particular importance for the Mediterranean Sea, offering valuable insights into the interactions between native and invasive species. Continuous monitoring of these communities and the integration of ecological and sedimentological approaches will be crucial for understanding the future evolution of Mediterranean benthic ecosystems and for guiding potential management and conservation strategies. Given the limited possibilities for controlling or eradicating invasive species of small sizes, long-term monitoring and conservation of vegetated substrates are essential tools for mitigating their ecological impact. The results obtained highlight the urgent need to integrate benthic foraminifera data into broader marine monitoring programs and to use diversity indices as early warning indicators of bioinvasion in Mediterranean coastal ecosystems.

## Figures and Tables

**Table 1:** Geographic coordinates, bathymetry, and type of sample collected at each sampling site. Two values for temperature and salinity are reported; * indicate our measurements during sample collection, while the other value is from [48].

Sites	Latitude	Longitude	Sites	SST (°C)	SSS (‰)	Depth (m)	Sample Type
Gadir	36°48′42″ N	12°01′32″ E	PANT 24-3-1A	24 *–22.6	37 *–37.5	5	*P. oceanica*’s rhizomes
			PANT 24-3-1			6.5	*P. oceanica*’s rhizomes
			PANT 24-3-2			10.5	*P. oceanica*’s rhizomes
			PANT 24-3-3			17.5	*P. oceanica*’s rhizomes
Cala Tramontana	36°47′54″ N	12°02′52″ E	PANT 24-4-1	24 *–22.8	37 *–37.5	7	brown algae *Halopteris scoparia*
			PANT 24-4-2			9	*P. oceanica*’s rhizomes
			PANT 24-4-3			20	brown algae *Halopteris scoparia*
Balata dei Turchi	36°44′10″ N	12°01′09″ E	PANT 24-5-1	24 *–22.9	37 *–37.5	4	brown algae *Halopteris scoparia*
			PANT 24-5-2			5	sediment

**Table 2:** Numbers and percentages of total living versus total dead foraminifera in the studied samples.

Sampling Sites	No. of Living Specimens	No. of Dead Specimens	No. of Total Specimens (Living + Dead)	Total Living Foraminifera (%)	Total Dead Foraminifera (%)
PANT 24-3-1A	111	0	111	100.00	0.00
PANT 24-3-1	296	0	296	100.00	0.00
PANT 24-3-2	218	0	218	100.00	0.00
PANT 24-3-3	118	31	149	79.19	20.81
PANT 24-4-1	216	13	229	94.32	5.68
PANT 24-4-2	251	15	266	94.36	5.64
PANT 24-4-3	342	19	361	94.74	5.26
PANT 24-5-1	240	3	243	98.77	1.23
PANT 24-5-2	209	81	290	72.07	27.93

**Table 3:** Percentages of living foraminiferal species.

Benthic Foraminiferal Species	PANT 24-3-1A	PANT 24-3-1	PANT 24-3-2	PANT 24-3-3	PANT 24-4-1	PANT 24-4-2	PANT 24-4-3	PANT 24-5-1	PANT 24-5-2
*Adelosina* sp. 1	0.90	0.00	0.00	0.00	0.00	3.59	0.00	0.00	0.00
*Adelosina* sp. 2	0.00	0.00	0.00	0.00	0.93	0.00	0.00	0.00	0.00
*Amphisorus hemprichii*	0.00	0.34	0.00	0.00	0.00	0.00	0.00	0.00	0.00
*Amphistegina lobifera*	21.62	59.12	66.97	20.34	27.78	23.51	7.31	39.17	69.86
*Amphistegina lessonii*	4.50	7.09	10.55	31.36	18.06	9.16	19.59	28.33	6.70
*Amph.* morphotype alfa	0.00	1.35	0.00	1.69	0.93	0.40	0.00	0.00	0.48
*Asterigerinata mamilla*	6.31	1.35	0.46	4.24	3.70	2.79	0.00	0.00	0.48
*Bolivina catanensis*	0.00	0.00	0.00	0.00	0.00	0.40	0.00	0.00	0.00
*Cribroelphidium* sp.	0.00	0.00	0.00	0.00	0.00	0.80	0.29	0.00	0.00
*Cyclocibicides vermiculatus*	0.00	0.00	0.00	4.24	0.93	0.00	0.00	0.00	0.00
*Cymbaloporetta squammosa*	2.70	0.68	0.00	0.00	1.39	0.00	0.00	0.00	0.00
*Laevipeneroplis* sp.	8.11	0.00	0.00	0.00	1.39	0.00	0.00	0.42	0.00
*Lachlanella variolata*	2.70	0.00	0.00	0.00	0.00	0.00	0.00	0.00	0.00
*Lobatula lobatula*	4.50	1.01	0.92	0.00	1.39	0.00	21.93	0.00	0.96
*Miliolinella subrotunda*	18.92	11.49	5.96	3.39	8.33	5.18	13.74	7.08	2.39
*Peneroplis pertusus*	10.81	9.12	3.21	3.39	5.56	13.55	0.00	16.25	6.22
*Peneroplis planatus*	0.90	0.00	0.92	1.69	0.93	1.99	0.88	0.00	2.39
*Planorbulina acervalis*	1.80	0.68	1.83	5.08	0.93	3.59	8.48	0.42	0.00
*Quinqueloculina* sp. 1	0.90	1.35	0.92	5.08	3.70	7.17	2.34	0.83	1.44
*Quinqueloculina* sp. 2	4.50	0.34	2.29	0.00	2.78	7.57	1.75	2.08	2.39
*Quinqueloculina* sp. 3	0.00	0.00	0.00	0.00	0.46	0.00	0.00	0.00	0.00
*Quinqueloculina* sp. 4	0.00	0.00	0.00	0.00	0.00	0.00	2.34	0.00	0.00
*Rosalina bradyi*	0.00	0.00	0.00	0.00	0.00	0.80	0.00	0.00	0.00
*Rosalina obtusa*	6.31	2.03	1.83	0.00	3.70	4.78	11.70	2.08	0.96
*Spiroloculina excavata*	0.00	0.00	0.00	0.00	0.00	1.99	0.00	0.00	0.00
*Spiroloculina* sp.	0.00	0.00	0.00	0.00	0.46	1.99	0.00	0.00	0.00
*Textularia pala*	1.80	3.04	1.38	16.10	12.50	7.57	4.39	2.50	5.74
*Tretomphalus bulloides*	0.00	0.00	0.00	0.00	0.46	0.00	0.00	0.00	0.00
*Vertebralina striata*	2.70	1.01	2.75	3.39	3.70	3.19	5.26	0.83	0.00

## Data Availability

Data presented in this study have been included in the manuscript. Further inquiries can be directed to the Corresponding Author (C.C.).
